# Homologous recombination repair deficiency (HRD) testing in newly diagnosed advanced-stage epithelial ovarian cancer: A Belgian expert opinion

**DOI:** 10.52054/FVVO.14.2.024

**Published:** 2022-07-01

**Authors:** I Vergote, H Denys, S Altintas, J Kerger, J-F Baurain, V Bours, S Henry, K Van de Vijver, D Lambrechts, C Gennigens

**Affiliations:** Department of Gynaecological Oncology, University Hospitals Leuven, Leuven Cancer Institute, Herestraat 49, 3000 Leuven, Belgium; Department of Medical Oncology, Ghent University Hospital, C. Heymanslaan 10, 9000 Ghent, Belgium; Multidisciplinary Oncologic Centre Antwerp (MOCA), Antwerp University Hospital, 2650 Edegem, Belgium; Department of Medical Oncology in the SORMN, CHU UCL Namur, Place Louise Godin 15, 5000 Namur, Belgium; Jules Bordet Institute, Brussels University Hospital, Université Libre de Bruxelles, Boulevard de Waterloo 121, 1000 Brussels, Belgium; Department of Medical Oncology, Cliniques Universitaires Saint-Luc, UCLouvain, avenue Hippocrate 10, 1200 Brussels, Belgium; Department of Human Genetics, CHU Liège, 4000 Liège, Belgium; Department of Pathology, Ghent University Hospital, C. Heymanslaan 10, 9000 Ghent, Belgium; Department of Pathological Anatomy, Antwerp University Hospital, Universiteitsplein 1, 2610 Wilrijk, Belgium; Laboratory of Translational Genetics (VIB-KU Leuven), ON IV Herestraat 49 - box 912, 3000 Leuven, Belgium; Department of Medical Oncology, Liège University Hospital, Avenue de l’Hôpital 1, 4000 Liège, Belgium

**Keywords:** HRR deficiency, genomic instability, advanced ovarian cancer, PARPi

## Abstract

Ovarian cancer (OC) has a poor prognosis as most patients present with non-specific symptoms and the disease is mostly diagnosed at advanced stages. Approximately 90% of cases are classified as epithelial OC (EOC), a category comprising histologically and molecularly distinct tumours. Identifying reliable biomarkers and employing personalised therapies in OC subgroups is crucial for battling the disease. EOCs are often characterised by homologous recombination repair deficiency (HRD), frequently caused by inactivation of the breast cancer susceptibility (BRCA) genes. These findings have led to the development of poly- (adenosine diphosphate [ADP])- ribose polymerase inhibitors (PARPi), which are synthetically lethal to HRD tumour cells. Both patients with HRD and non-HRD tumours can benefit from PARPi therapy in the recurrent setting. Moreover, recent phase III trials in patients with newly diagnosed advanced-stage OC have demonstrated greater clinical benefit from PARPi in treating HRD than non-HRD tumours. These findings offer new opportunities for the use of PARPi as maintenance therapy after first-line chemotherapy based on the presence of HRD. In the current article, we provide recommendations for HRD testing and treatment of patients with newly diagnosed advanced-stage EOC.

## Introduction

Ovarian cancer (OC) is among the most lethal gynaecological cancers in the United States and Europe. Because symptoms are non-specific, OC diagnosis is usually delayed at the more advanced stages (International Federation of Obstetrics and Gynaecology [FIGO] stage III and IV), (Colombo et al., 2019; Vergote et al., 2020). OC comprises a heterogeneous group of diseases with various histological subtypes, differentiation grades, and molecular characteristics (Lheureux et al., 2019). Approximately 90% of OCs are of epithelial origin (EOC), the most common type of which is high grade serous carcinoma (HGSOC) (Vergote et al., 2020).

**Figure 1:**
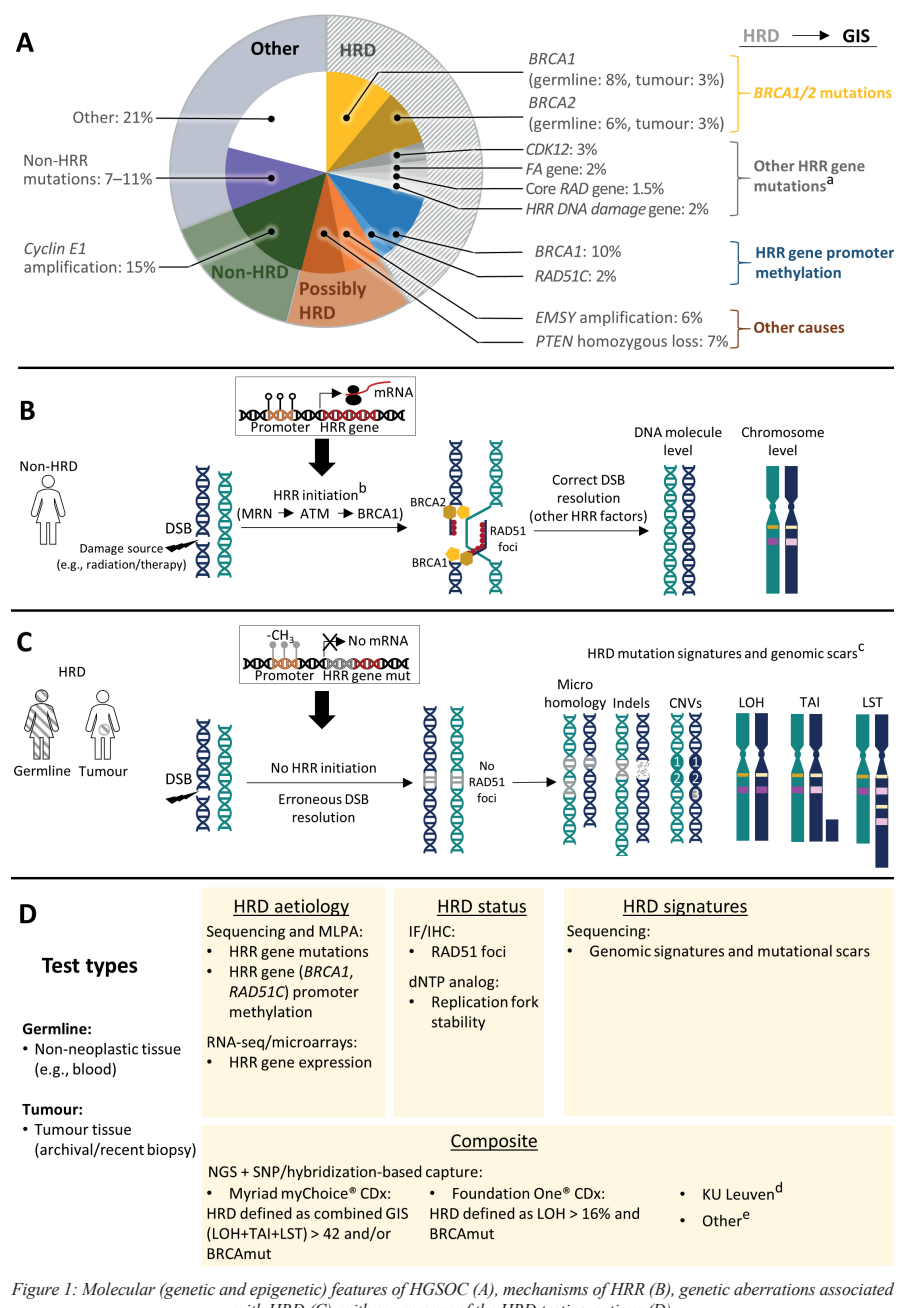
Molecular (genetic and epigenetic) features of HGSOC (A), mechanisms of HRR (B), genetic aberrations associated with HRD (C), with a summary of the HRD testing options (D) ^*a^ Not all mutations have been linked to an HRD phenotype; ^b^A simplified view on the DNA repair pathway is indicated, whereby the major steps involve sequential recruitment of the MRN complex, the ATM and BRCA1 to the break spot; ^c^The mutation signatures and genomic scars are represented according to authors’ idea; ^d^The KU Leuven assay is in development; ^e^Other HRD tests are also in development but have not yet been validated versus clinical outcomes. ATM, ataxia telangiectasia mutated serine/threonine kinase; BRCA1 and BRCA2, breast cancer susceptibility gene; BRCAmut, genotype without a functional BRCA1 and or BRCA2 allele; CDK12, cyclin dependent kinase 12; -CH3, methyl group, indicating histone methylation; CNVs, copy number variations; dNTP, deoxyribonucleotide triphosphate; DSB, double strand DNA break; EMSY, BRCA2 interacting transcriptional repressor; FA, Fanconi anaemia; gBRCAmut and tBRCAmut, germline and tumour BRCA1/2 mutations; GIS, genome instability score; HGSOC, high-grade serous ovarian cancer; HRD, homologous recombination DNA repair deficiency; HRR, homologous recombination DNA repair; IF, immunofluorescence; IHC, immunohistochemistry; Indels, insertions/deletions; LOH, loss of heterozygosity; LST, large-scale state transitions; MLPA, multiplex ligation-dependent probe amplification; mRNA, messenger RNA; MRN, Mre11-Rad50-Nbs1 complex; NGS, next generation sequencing; PTEN, phosphatase and tensin homolog; RAD, RecA-like protein; RNA-seq, RNA sequencing; SNPs, single nucleotide polymorphisms; TAI, telomeric allelic imbalance. Panel A was adapted from (Konstantinopoulos et al., 2015).

EOC is genetically heterogeneous (Lheureux et al., 2019). Chromosomal instability and inactivation of tumour suppressor genes are common in HGSOC (also referred to as tubo-ovarian HGSOC) (The Cancer Genome Atlas Research Network, 2011), while non-HGSOC is primarily characterised by recurrent tumour mutations (Vergote et al., 2020). As illustrated in Figure 1A, approximately half of HGSOC cases are deficient for the homologous recombination deoxyribonucleic acid (DNA) repair (HRR) pathway (The Cancer Genome Atlas Research Network, 2011).

### Homologous recombination DNA repair deficiency (HRD)

The HRR pathway is a low-error mechanism to repair double stranded DNA breaks (DSBs) caused by endogenous (e.g., DNA replication defects) or exogenous (e.g., chemotherapeutic agents) factors (Fuh et al., 2020). Cell-culture-based experiments indicate that dozens of DSBs are likely to occur in human cells daily (Fuh et al., 2020). The HRR pathway is active during the synthesis and gap 2 cell cycle phases, when an intact homologous chromosome is available as a repair template. Following DSB detection, several proteins are recruited to the damaged DNA site to correctly repair the break (Figure 1). Among the best known and characterised proteins are breast cancer susceptibility type 1 and 2 (BRCA1 and BRCA2), ataxia-telangiectasia mutated (ATM), the MRN complex (consisting of meiotic recombination 11 [MRE11], RecA-like protein 50 [RAD50] and Nijmegen breakage syndrome protein 1 [NBS1]), and RAD51 (Fuh et al., 2020). Of particular interest in EOC is also poly-(adenosine diphosphate [ADP])-ribose polymerase (PARP), involved in single strand DNA break repair (Ray Chaudhuri and Nussenzweig, 2017).

HRR deficiency (HRD) results from malfunction of the HRR pathway, caused either through genetic (e.g., pathogenic mutations) or epigenetic inactivation (e.g., promoter methylation) or downregulated expression of HRR-related genes (Fuh et al., 2020). Both inherited (germline) and de novo (tumour) HRD-causing aberrations have been linked to EOC (Miller et al., 2020). Homozygous inactivation of BRCA1 and BRCA2 genes (collectively denoted the BRCAmut genotype) has been described in most EOC subtypes, and in up to 40.0% of unselected OC patients (Hennessy et al., 2010; The Cancer Genome Atlas Research Network, 2011; Swisher et al., 2017; Haunschild and Tewari, 2021). BRCAmut EOCs respond well to therapy with PARP inhibitors (PARPi), with an improved progression-free and overall survival (PFS and OS) (Moore et al., 2018; Coleman et al., 2019; González- Martín et al., 2019; Ray-Coquard et al., 2019). Indeed, these mutations are synthetically lethal in combination with DNA repair inhibition (Vergote et al., 2020). Additionally, patients harbouring germline or tumour BRCAmut (gBRCAmut or tBRCAmut) alterations are sensitive to platinum- based chemotherapy (Colombo et al., 2019; Vergote et al., 2020). Some tumours harbour functional BRCA1/2 genes (denoted BRCAwt) but still exhibit BRCAmut-like patterns of genomic instability. Indeed, non-BRCA1/2 DNA repair defects (e.g., mutations in other HRR-related genes) may confer BRCAmut-like drug sensitivities (e.g., to platinum- based chemotherapy and PARPi) (Lheureux et al., 2019; Fuh et al., 2020). Approximately 30% of HGSOCs have non-BRCA1/2 genomic alterations resulting in HRD (Konstantinopoulos et al., 2015). Mutations of non-BRCA1/2 HRR-related genes are also included in some common genomic testing panels for (hereditary) OC (Haunschild and Tewari, 2021).

The consequences of HRD are varied (Fuh et al., 2020), as illustrated in Figure 1B and 1C. If HRR is impaired, the damaged DNA is repaired by more error-prone mechanisms. This leads to genomic instability reflected in genetic alterations of variable sizes, including a specific set of single nucleotide polymorphisms (SNPs), insertions and deletions (collectively termed indels, up to 1 kilobase [kb] in size) flanked by short tandem repeats, overlapping microhomologies (short, identical sequence stretches at DNA breakpoints) (Nik-Zainal et al., 2012), and copy number variations (CNVs; indels larger than 1 Kb that alter gene expression) (Haunschild and Tewari, 2021). Larger rearrangements also occur and include loss of heterozygosity (LOH), telomeric allelic imbalance (TAI) and large-scale state transitions (LST), jointly referred to as “genomic scars” (Figure 1). LOH occurs if an entire allele is lost due to faulty DNA repair (e.g., through a larger indel), while TAI and LST involve loss of larger chromosomal regions (≥10─15 mega base [Mb]) (Watkins et al., 2014; Haunschild and Tewari, 2021). Genomic instability and scarring patterns are specific for the defective DNA repair pathway.

### Molecular tests of HRD positivity

HRD testing should ideally be performed to establish the genetic profile of the tumour, estimate patient prognosis, and guide appropriate therapy (Haunschild and Tewari, 2021). Germline mutations of HRR-related genes, including BRCA1 and BRCA2, are established hereditary risk factors for developing multiple malignancies. Germline and tumour BRCA1/2 status are highly concordant (i.e., most tBRCAmut alterations are germline) (Vergote et al., 2020), although BRCA1/2 mutations detected in 5%─7% patients with HGSOC are identified in the tumour, but not through germline testing (Callens et al., 2021). Moreover, BRCA1/2 mutations are known predictors of OC response to PARPi (Miller et al., 2020), while the evidence for other HRR-related gene mutations in conferring PARPi sensitivity is still controversial, especially in the first-line setting. Additionally, it is essential to identify good predictors of the OC biological status as HRD or non-HRD (Haunschild and Tewari, 2021). Various genomic and biochemical tests have been developed to check for HRD positivity in human tissue (Figure 1D).

### Testing approaches

Genomic HRD tests detect germline and tumour mutations of HRR-related genes, as well as genomic scars indicative of HRD (Fuh et al., 2020; Miller et al., 2020). Specific tests are needed to provide a readout of the genomic scars, such as genome wide LOH (e.g., FoundationOne® CDx) or a multicomponent genomic instability score (GIS) (e.g., Myriad myChoice® CDx). According to genomic tests, tumour samples are classified as HRD if they bear a BRCAmut and/or are positive for genomic scars (i.e., genomic instability score above a pre-defined threshold). The gain from sequencing readouts is thus two-fold as both the genetic causes and consequences of HRD may be identified. For clinical purposes, several next generation sequencing (NGS) tests have been validated. The NGS method enables parallel sequencing of thousands of pre- defined genomic loci with high sensitivity and accuracy (Haunschild and Tewari, 2021).

Non-neoplastic tissue (e.g., blood, saliva) is used for germline tests, while tumour tests are conducted on freshly frozen or formalin-fixed and paraffin- embedded (FFPE) tumour tissue. Tumour-based assays are technically more challenging compared to germline testing due to histological (e.g., low tumour cell content) and clonal heterogeneity of tumour tissue samples. Major factors for the success of sequencing approaches are sampling of sufficient high-quality material, and optimal tumour cell content (usually at least 30%) (Miller et al., 2020). Also, OC tumours are known to genetically change over time, in terms of tBRCAmut status and possible BRCA1/2 reversion mutations, but their larger genomic scar patterns detected by genomic instability testing remain quite stable over time (Patel et al., 2018).

Aside from the sequencing based HRD profiling tests, different molecular, biochemical, and cytological assays have been described to further characterise tumour HRD status. One of these is the RAD51 foci assay, which provides a functional readout of HRD. RAD51 normally accumulates at DSBs during functional HRR, so the impaired formation of RAD51 foci is the reflection of HRD (Ceccaldi et al., 2016). HRD scores based on functional RAD51 foci assays have been reported to correlate with tumour sensitivity to chemotherapy, PARPi response, and overall survival (Fuh et al., 2020), but have not yet been reported in major prospective randomised phase III studies. Limitations of the RAD51 foci assays are the high technicality, lack of automation for foci counting, and the complex HRD score calculation.

Two recent review articles provide a detailed overview of additional but clinically still non- validated methods, which include gene expression profiling, promoter methylation, and replication fork stalling assays (Fuh et al., 2020; Haunschild and Tewari, 2021).

#### Genomic HRD tests available in Belgium

Several tests were recently recommended by the Personalised Medicine Commission (ComPerMed) (Belgian Cancer Registry, 2018) and are summarised in Table I. The commercially available assays (from Myriad Genetics and Foundation Medicine) are available worldwide but are not yet reimbursed in the Belgian health system and were so far conducted only in the context of clinical trials.

**Table I:** Overview of diagnostic genetic HRD tests recommended by ComPerMed and available in Belgium.

Level	Test (Platform/Method)	Specimen	Genes (for OC)	Readout	Processing time (from sample receipt)	Clinical utility/reference
Germline	BRCA1/2-containing gene panels (Illumina [NovaSeq] and other)	Whole blood in EDTA (10─20 mL); saliva (2 mouth swabs); 15─30 mg tissue biopsy; 50─100 µg gDNA	5─27 (BRCA1, BRCA2, ATM, CDH1, CHEK2, RAD50, RAD51C, RAD51D, NBN, MRE11A, p53, BRIP1, MLH1, MSH2, MSH6, PALB2, PTEN)	SNPs, indels, CNVs	6 weeks─6 months	(orpha.net, 2021)^a^
	Myriad BRAC-Analysis® CDx	Whole blood in EDTA (~ 7 mL)	2 (BRCA1, BRCA2)	SNPs, indels, large deletions and duplications	Not specified	SOLO-1 (Moore et al., 2018)
Tumour	FoundationOne® CDx (Illumina)	FFPE tissue block or 10 unstained slides with minimum 20.0% cells of tumour origin; minimum sample surface area: 25 mm^2^	324 (BRCA1, BRCA2, ATM, ATR, CHEK1, CHEK2, RAD50, RAD51B, RAD51C, RAD51D, NBN, MRE11A, p53, BARD1, BRIP1, MLH1, PARP1, PARP2)^b^	SNPs, CNVs, indels, selected genomic rear-rangements, LOH (scored as percentage in tumour genome- low: <16.0%; high: ≥16.0%), signatures of non-HRR	≤10 days	ARIEL2 (Swisher et al., 2017), ARIEL3 (Coleman et al., 2017)
	FoundationOne Liquid® CDx (Illumina NovaSeq 6000)	8.5 ml whole blood, before or 2 weeks after chemotherapy	324^c^	SNPs, CNAs, indels, selected genomic rearrangements	≤10 days	
	Myriad my-Choice® CDx (Illumina)	FFPE tissue block or 10 unstained slides with minimum sample surface area: 25 mm^2^	2 (BRCA1, BRCA2)	SNPs, indels, large deletions and duplications, GIS score (GIS = LOH + TAI + LST score; HRD if GIS ≥42 and/or BRCAmut)	≤2 weeks	PRIMA, PAOLA-1, VELIA, NOVA, QUADRA (Swisher et al., 2017; Coleman et al., 2019; González-Martín et al., 2019; Moore et al., 2019; Ray-Coquard et al., 2019)
	BRCA1 and BRCA2 tumour analysis (Illumina and other)	FFPE or fresh tumour tissue (≥ 10% of tumour cell content);	2 (BRCA1, BRCA2)	SNPs, indels	1─3 months	(orpha.net, 2021)
	KU Leuven (Illumina)	FFPE tumour tissue	90000 genome-wide SNPs (BRCA1, BRCA2, RAD51C, RAD51D, p53, BRIP, BARD, PALB2 and BLM)	Capture-based assay; SNPs, genomic scars	Not specified	PAOLA-EN-GOT-ov25 (Pujade-Lauraine et al., 2021b)

^a^ The assays listed in orpha.net refer to summaries of all genetic testing methods for ovarian cancer patients at different centres in Belgium; ^b^For large gene panels, only select HRR-related genes are listed; ^c^The list is the same as for FoundationOne® CDx assay. ATM, ataxia telangiectasia mutated gene; ATR, ataxia telangiectasia and Rad3 related gene; BARD1, BRCA1 associated really interesting new gene (RING) domain 1 gene; BLM, Bloom syndrome protein gene; BRCA1/2, breast cancer susceptibility genes 1 and 2; BRIP, BRCA1 Interacting Protein C- terminal helicase; CDH1, cadherin 1 gene; CDK12, cyclin dependent kinase 12 gene; CHEK1/2, checkpoint kinase 1 and 2 genes; CNVs, copy number variations; EDTA, ethylene di-amine tetra-acetic acid; FANCA/L, Fanconi anaemia complementation group A and L genes; FFPE, formalin-fixed paraffin-embedded; gDNA, genomic DNA; GIS, genomic instability score; HRR, homologous recombination repair; indels, insertions and deletions; LOH, loss of heterozygosity; LST, large-scale state transitions; MLH1, MutL-homologue 1 gene; MRE11A, meiotic recombination 11 homologue A gene; MSH2/6, MutS-homologue genes 2 and 6; NBN, nibrin gene; OC, ovarian cancer; PALB2, partner and localiser of BRCA2 gene; PARP1/2, poly-(adenosine diphosphate [ADP])-ribose polymerase 1 and 2 genes; PTEN, phosphatase and tensin homologue gene; RAD50/51B/51C/51D/54L, RecA-like protein 50, 51B, 51C, 51D, and 54L genes; SNPs, single nucleotide polymorphisms; TAI, telomeric allelic imbalance.

#### Clinical utility of HRD testing

Current HRD tests measure a genotype indicative of HRD. A correlation between HRD test scores and PARPi treatment benefit is a common criterion to evaluate if a particular test score is clinically meaningful (Miller et al., 2020; Haunschild and Tewari, 2021).

Until recently, most established data on the use of BRCA status for PARPi treatment decisions came from studies in relapsed OC setting. In platinum- sensitive relapsed OC, PARPi treatment is active as maintenance monotherapy also in patients with tBRCAwt tumours, although with lower benefit than in tBRCAmut carriers (Mirza et al., 2016; Coleman et al., 2017). Two phase II studies in relapsed setting (ARIEL2 and QUADRA) found that tumour HRD status, determined by either the genomic LOH score (FoundationOne® CDx) or combined GIS (Myriad myChoice®CDx), was a predictor of OS with PARPi treatment (Swisher et al., 2017; Moore et al., 2019).

Furthermore, more recent clinical trials provide evidence that HRD genomic profiling is an important determinant of PARPi therapy response in newly diagnosed advanced stage compared to relapsed OC. These trials also highlight the need for reliable and standardised profiling of advanced-stage OC patients, to identify those who may significantly benefit from targeted PARPi therapy. The four published randomised phase III clinical trials investigating PARPi treatment in newly diagnosed advanced-stage OC are: PRIMA-ENGOT-ov26, PAOLA-1-ENGOT-ov25, VELIA and SOLO-1 (Moore et al., 2018; Coleman et al., 2019; González- Martín et al., 2019; Ray-Coquard et al., 2019). All were well-designed, double-blind, placebo- controlled trials, including only stage III and IV OC patients. Direct comparison between these trials is complicated because of their distinct designs and analysis methods (Table II). A common outcome of these trials was the statistically significant increase in median PFS of patients with an advanced-stage EOC and a confirmed HRD status (presence of BRCAmut and/or genomic scars) after PARPi treatment.

**Table II:** Key clinical trials of first-line PARPi treatment enrolling patients with newly diagnosed FIGO stage III and IV OC.

Clinical trial	Patients (number)	Treatment^a^ (randomisation)	HRD status	PFS	OS	HR for disease progression/death (95% CI)
SOLO-1 (Moore et al., 2018; Banerjee et al., 2020)	HGS or endometrioid OC, primary peritoneal or fallopian tube cancer (or combination) (391)	Maintenance olaparib: placebo (2:1)	BRCAmut	Olaparib: 56 months placebo: 14 months		0.30 (0.23–0.41)
PAOLA-1-ENGOT-ov25 (Ray-Coquard et al., 2019)	HGS or endometrioid OC, primary peritoneal or fallopian tube cancer. Patients in complete or partial response after platinum-based chemotherapy+BEV (806)	Maintenance olaparib+BEV: BEV alone (2:1)	Overall^b^ BRCAmut HRD (Myriad myChoice®CDx GIS≥42 or tBRCAmut)	Overall: • olaparib+BEV: 22 months • BEV alone: 17 months BRCAmut: • olaparib+BEV: 37 months • BEV alone: 22 months HRD: • olaparib+BEV: 37 months • BEV alone:18 months Non-HRD or HRD unknown: • olaparib+BEV: 17 months • BEV alone:16 months		Overall: 0.59 (0.49–0.72) BRCAmut: 0.31 (0.20–0.47) HRD: 0.33 (0.25─0.45) Non-HRD or HRD unknown: 0.92 (0.72─1.17)
PRIMA-ENGOT-ov26 (González-Martín et al., 2019)	OC, peritoneal or fallopian tube cancer. Patients in complete or partial response after platinum-based chemotherapy (733)	Maintenance (for 36 months or until disease progression) niraparib : placebo (2:1)	Overall^b^ HRD (Myriad myChoice®CDx GIS≥42 and/or BRCAmut)	Overall: niraparib: 14 months • placebo: 8 months HRD: • niraparib: 22 months • placebo: 10 months Non-HRD: • niraparib: 8 months • placebo: 5 months	Overall at 24 months: • niraparib: 84% • placebo: 77% HRD at 24 months: • niraparib: 91% • placebo: 85% Non-HRD at 24 months: • niraparib: 81% placebo: 59%	Overall: 0.62 (0.50–0.76) HRD: 0.43 (0.31–0.59) Non-HRD: 0.68 (0.49─0.94)
VELIA (Coleman et al., 2019)	Previously untreated HGSOC, peritoneal or fallopian tube cancer (1140)	veliparib throughout (chemotherapy and maintenance): veliparib combination (only chemotherapy): control (placebo thoughout) (1:1:1)	Overall^b^BRCAmut HRD (Myriad myChoice®CDx GIS≥33 and/or BRCAmut)	Overall: • veliparib throughout: 24 months • veliparib combination: 15 months • control: 17 months BRCAmut: • veliparib throughout: 35 months • veliparib combination: 21 months • control: 22 months HRD: • veliparib throughout: 32 months • veliparib combination: 18 months • control: 21 months Non-HRD: • veliparib throughout: 15 months • veliparib combination: 13 months • control: 12 months		Overall (versus control): • veliparib throughout: 0.68 (0.56–0.83) • veliparib combination: 1.07 (0.90–1.29) BRCAmut (versus control): • veliparib throughout: 0.44 (0.28–0.68 • veliparib combination: 1.22 (0.82–1.80) HRD (versus control): • veliparib throughout: 0.57 (0.43–0.76 • veliparib combination: 1.10 (0.86–1.41) Non-HRD (versus control): • veliparib throughout: 0.81 (0.60–1.09 • veliparib combination: 1.04 (0.78–1.39)

^a^Treatment refers to administration of poly-(adenosine diphosphate [ADP])-ribose polymerase inhibitor (PARPi) or corresponding control; ^b^Overall population includes all patients in a trial, regardless of their HRD status. BEV, bevacizumab; BRCAmut, genotype with a homozygous inactivation of breast cancer susceptibility genes; CI, confidence interval; FIGO, International Federation of Obstetrics and Gynaecology; HGS, high-grade serous; HGSOC, high-grade serous ovarian cancer; HR, hazard ratio; HRD, homologous recombination repair deficiency; OC, ovarian cancer; OS, overall survival; PFS, progression-free survival.

Additional analyses on patient tumour samples from the PAOLA-1-ENGOT-ov25 trial using the Myriad myChoice® CDx assay showed that mutations in non-BRCA HRR-related genes were present in 3.7%–9.8% of patients (Pujade-Lauraine et al., 2021a; Pujade-Lauraine et al., 2021b). Unlike the HRD status, none of the tested non-BRCA gene panels were predictive of prolonged PFS in patients treated with olaparib and bevacizumab (BEV) (Pujade-Lauraine et al., 2021a). Mutations in some of these genes (e.g., BRIP1, RAD51C, RAD51D, PALB2) can result in HRD and are known to increase the risk of developing OC (Vergote et al., 2022). However, despite being useful for preventive familial screening and clinical research, they cannot be used as predictive markers of response to PARPi- containing therapies (Pujade-Lauraine et al., 2021a; Vergote et al., 2022).

Importantly, the PRIMA-ENGOT-ov26, PAOLA-1-ENGOT-ov25, and VELIA trials confirmed that gBRCA, tBRCA, and HRD status (as determined by the multicomponent GIS) are relevant determinants of PARPi response. Of note, GIS-based HRD scoring may need to be adapted due to overlapping hazard ratios in subgroup analyses. Alternatively, new HRD tests with adapted cut-offs, such as the recently developed assay of KU Leuven and collaborators, may relate a patient’s HRD status with predicted response to targeted therapy (Loverix et al., 2022). All these data are in favour of reimbursing BRCA1/2 and genomic HRD testing to identify patients who can benefit most from the PARPi therapy.

Aside from PARPi, it is important to consider inclusion of BEV in the treatment algorithm of OC. In clinical trials, BEV has demonstrated benefits in terms of PFS when administered in first line with standard chemotherapy or in relapsed disease regardless of platinum sensitivity (Vergote et al., 2020). BEV-associated improvements in OS are currently restricted to the ICON7 and GOG- 218 trial findings in high-risk and advanced-stage patients, respectively (Perren et al., 2011; Norquist et al., 2018). There are still no reliable predictive biomarkers to inform decisions on which patients should or should not receive BEV (Colombo et al., 2019).

#### ESMO and ESGO guidelines on HRD testing

Supported by recent data in patients with newly diagnosed advanced-stage EOC (Coleman et al., 2019; González-Martín et al., 2019; Moore et al., 2019; Ray-Coquard et al., 2019), the European Society for Medical Oncology (ESMO) and European Society of Gynaecological Oncology (ESGO) concede that BRCA1/2 status is a good predictor of patients’ response to PARPi and the extent to which a patient can benefit from such a treatment (Colombo et al., 2019; Miller et al., 2020). Furthermore, it was advised that all patients with HGSOC should be tested for mutations in BRCA1/2, and possibly other HRR-related genes (e.g., RAD51C, RAD51D, BRIP1, and PALB2). In the setting of first-line maintenance treatment, ESMO in 2020 recommended that gBRCAmut and tBRCAmut, as well as genomic scars should be routinely tested to identify HGSOC patients who may benefit from PARPi treatment (Miller et al., 2020).

Belgian consensus on HRD testing and treatment of newly diagnosed advanced-stage EOC

**Figure 2:**
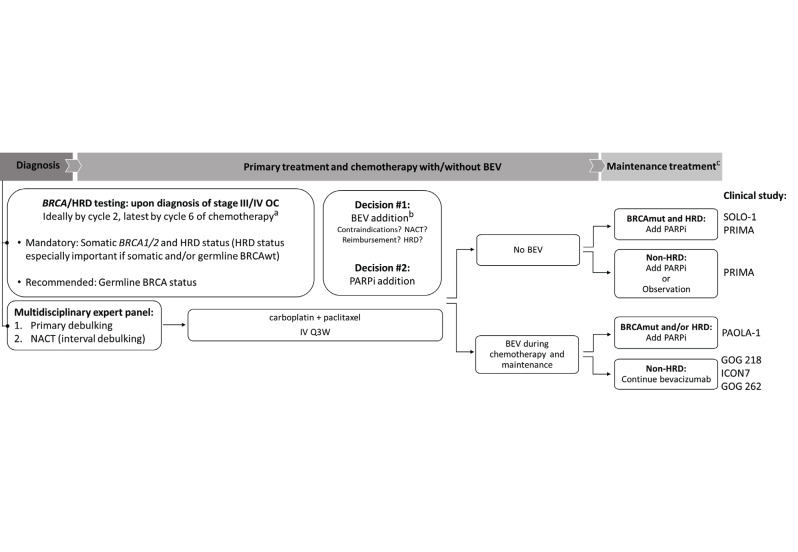
Consensus of Belgian physicians on HRD testing and treatment algorithm for newly detected advanced-stage epithelial ovarian cancer. ^*a^ genetic testing results should be known before the 2nd cycle of chemotherapy for patients who underwent primary debulking and who are candidates for bevacizumab treatment; ^b^reimbursed only for stage IV carcinoma in Belgium (situation in November 2021); ^c^PARPi reimbursement criteria were not extended to accommodate HRD testing outcomes (Belgium, November 2021). BEV, bevacizumab; BRCA, breast cancer susceptibility gene; BRCAmut, genotype without a functional BRCA1 and/or BRCA2 gene; BRCAwt, normal (wild-type) genotype; HRD, homologous recombination DNA repair deficiency; IV Q3W, intravenous administration every three weeks; NACT, neo-adjuvant chemotherapy; OC, ovarian cancer; PARPi, poly- (adenosine diphosphate [ADP])-ribose polymerase inhibitor. The referenced clinical trials are SOLO-1 (Moore et al., 2018), PRIMA (González-Martín et al., 2019), PAOLA-1 (Ray-Coquard et al., 2019), GOG 218 (Burger et al., 2011), GOG 262 (Chan et al., 2016), and ICON7 (Perren et al., 2011).

Given its relevance to the therapeutic response, we hereby advocate for early and reliable HRD testing in EOC patients, to ensure optimal and timely treatment decisions. We focus on clinical and practical recommendations for Belgian physicians and provide a consensus expert opinion on the decision-making process for newly diagnosed stage III─IV EOC (Figure 2). The presented recommendations are based on the available clinical research evidence and outline an ideal-case scenario for a maximised benefit of HRD testing and proposed therapies, acknowledging that not all testing and treatment options may be accessible for each patient. The clinical experience and the presented consensus opinion are similar to European guidelines and expert panel outcomes published in the last years (Colombo et al., 2019; Miller et al., 2020; Vergote et al., 2022).

As per recent Belgian regulatory decisions, PARPi reimbursement criteria were not extended to accommodate HRD testing outcomes (“Substantiated final proposal from the Medicines Allowance 2021/155-0154124504 Commission for an application to amend Lynparza’s reimbursement modalities”). The implementation of below recommendations will therefore depend on the patient’s individual status and diagnosis and future available treatment options.

PARPi (olaparib, rucaparib, niraparib) and BEV are approved by the European Medicines Agency (EMA) as targeted therapies for OC (Vergote et al., 2020). Details on reimbursement policies of these medicines for OC treatment in Belgium have been extensively summarised elsewhere (Belgian Cancer Registry, 2018; Vergote et al., 2020). EMA approved the combination of olaparib and BEV for use in patients with a confirmed HRD status, defined by BRCAmut and/or GIS (Myriad myChoice®CDx or another validated GIS) on FFPE tumour tissues (Belgian Cancer Registry, 2018; Vergote et al., 2020). Olaparib is reimbursed as a maintenance monotherapy in BRCAmut patients with newly diagnosed advanced-stage carcinoma, who partially or completely responded to platinum-based chemotherapy (Belgian Cancer Registry, 2018; Vergote et al., 2020). As of October 2021, niraparib will also be reimbursed for all patients with OC, except those with optimally debulked stage III OC (National Institute for Health and Disability Insurance (RIZIV/INAMI), 2021). In Belgium, BEV is only reimbursed in combination with chemotherapy to treat FIGO stage IV OC (Vergote et al., 2020).

Information on different aspects of HRD, including HRR gene mutations, GIS, LOH score, is complementary as it points to different patient subsets (Belgian Cancer Registry, 2018). NGS testing of non-BRCA1/2 mutations is not reimbursed in Belgium but can be accessed through certain trials. As discussed before (Vergote et al., 2020), it would be practical to implement “reflex” (guaranteed) BRCA1/2 genetic testing for all EOC patients as a standard pathology procedure.

Ideally, both gBRCA and tBRCA should be simultaneously tested at diagnosis. Therefore, NGS- based testing for tBRCA genotype and genomic instability scores should be conducted routinely for all advanced-stage EOC patients as soon as possible following diagnosis. This approach is also beneficial in view of current reimbursement policies, whereby simultaneous BEV and PARPi use is not reimbursed. Preferably, the genomic HRD test results should be known by the second cycle of chemotherapy (after 3 weeks of treatment) for patients undergoing primary debulking surgery (before the eventual start of BEV) or latest by the end of the sixth chemotherapy cycle (after 18 weeks of treatment) for all patients.

Collecting sample of sufficient quantity and quality and subjecting it to timely testing is key. Pathologists can prepare multiple samples following debulking surgery or tissue biopsy. It is important to consider the variable time between sample collection and complete test results, which depends on multiple steps: pathology diagnosis, referral waiting times for genetic counselling, shipment duration, and lab-specific test turnaround times (Haunschild and Tewari, 2021). The entire duration of the process can thus take two months on average (ORPHA, 2021).

As for maintenance treatment, PARPi are recommended for all except BRCAwt non-HRD patients who had received BEV during first-line chemotherapy (Figure 2). Observation only is an option for BRCAwt non-HRD patients who could not receive BEV in the first-line setting.

## References

[1] (2018). Cancer factsheet Ovarian Cancer ICD10: C56.

[2] Burger RA, Brady MF, Bookman MA (2011). Incorporation of bevacizumab in the primary treatment of ovarian cancer.. N Engl J Med.

[3] Callens C, Vaur D, Soubeyran I (2021). Concordance between tumor and germline BRCA status in high-grade ovarian carcinoma patients in the phase III PAOLA-1/ENGOT-ov25 trial.. J Natl Cancer Inst.

[4] Ceccaldi R, Rondinelli B, D’Andrea AD (2016). Repair Pathway Choices and Consequences at the Double-Strand Break.. Trends Cell Biol.

[5] Chan JK, Brady MF, Penson RT (2016). Weekly vs. Every-3-Week Paclitaxel and Carboplatin for Ovarian Cancer. N Engl J Med.

[6] Coleman RL, Fleming GF, Brady MF (2019). Veliparib with first-line chemotherapy and as maintenance therapy in ovarian cancer.. N Engl J Med.

[7] Coleman RL, Oza AM, Lorusso D (2017). Rucaparib maintenance treatment for recurrent ovarian carcinoma after response to platinum therapy (ARIEL3): a randomised, double-blind, placebo-controlled, phase 3 trial.. Lancet.

[8] Colombo N, Sessa C, du Bois A (2019). ESMO-ESGO consensus conference recommendations on ovarian cancer: pathology and molecular biology, early and advanced stages, borderline tumours and recurrent diseasedagger.. Ann Oncol.

[9] Fuh K, Mullen M, Blachut B (2020). Gynecol Oncol. Homologous recombination deficiency real-time clinical assays, ready or not.

[10] González-Martín A, Pothuri B, Vergote I (2019). Niraparib in patients with newly diagnosed advanced ovarian cancer.. N Engl J Med.

[11] Haunschild CE, Tewari KS (2021). The current landscape of molecular profiling in the treatment of epithelial ovarian cancer.. Gynecol Oncol.

[12] Hennessy BT, Timms KM, Carey MS (2010). Somatic mutations in BRCA1 and BRCA2 could expand the number of patients that benefit from poly (ADP ribose) polymerase inhibitors in ovarian cancer.. J Clin Oncol.

[13] Konstantinopoulos PA, Ceccaldi R, Shapiro GI (2015). Homologous recombination deficiency: exploiting the fundamental vulnerability of ovarian cancer.. Cancer Discov.

[14] Lheureux S, Braunstein M, Oza AM (2019). Epithelial ovarian cancer: evolution of management in the era of precision medicine.. CA Cancer J Clin.

[15] Loverix L, Vergote I, Busschaert P Predictive value of the Leuven HRD test compared with Myriad myChoice PLUS on 468 ovarian cancer samples from the PAOLA-1/ENGOT-ov25 trial.

[16] Miller RE, Leary A, Scott CL (2020). ESMO recommendations on predictive biomarker testing for homologous recombination deficiency and PARP inhibitor benefit in ovarian cancer.. Ann Oncol.

[17] Mirza MR, Monk BJ, Herrstedt J (2016). Niraparib maintenance therapy in platinum-sensitive, recurrent ovarian cancer.. N Engl J Med.

[18] Moore K, Colombo N, Scambia G (2018). Maintenance olaparib in patients with newly diagnosed advanced ovarian cancer.. N Engl J Med.

[19] Moore KN, Secord AA, Geller MA (2019). Niraparib monotherapy for late-line treatment of ovarian cancer (QUADRA): a multicentre, open-label, single-arm, phase 2 trial.. Lancet Oncol.

[20] Vergoedingsmodaliteiten: ZEJULA 100 mg - Niraparib. https://ondpanon.riziv.fgov.be/SSPWebApplicationPublic/nl/Public/ProductSearch.

[21] Nik-Zainal S, Alexandrov Ludmil B, Wedge David C (2012). Mutational Processes Molding the Genomes of 21 Breast Cancers.. Cell.

[22] Norquist BM, Brady MF, Harrell MI (2018). Mutations in homologous recombination genes and outcomes in ovarian carcinoma patients in GOG 218: an NRG Oncology/Gynecologic Oncology Group study.. Clin Cancer Res.

[23] Familial ovarian cancer https://www.orpha.net 2021

[24] Patel JN, Braicu I, Timms KM (2018). Characterisation of homologous recombination deficiency in paired primary and recurrent high-grade serous ovarian cancer.. Br J Cancer.

[25] Perren TJ, Swart AM, Pfisterer J (2011). A phase 3 trial of bevacizumab in ovarian cancer.. N Engl J Med.

[26] Pujade-Lauraine E, Brown J, Barnicle A (2021a). Homologous recombination repair mutation gene panels (excluding BRCA) are not predictive of maintenance olaparib plus bevacizumab efficacy in the first-line PAOLA-1/ENGOT-ov25 trial.. Gynecologic Oncology.

[27] Pujade-Lauraine E, Christinat Y, D’incalci M (2021b). 201 Homologous recombination deficiency testing in advanced ovarian cancer: description of the ENGOT HRD European initiative.. Int J Gynecol Cancer.

[28] Ray-Coquard I, Pautier P, Pignata S (2019). Olaparib plus bevacizumab as first-line maintenance in ovarian cancer.. N Engl J Med.

[29] Ray Chaudhuri A, Nussenzweig A (2017). The multifaceted roles of PARP1 in DNA repair and chromatin remodelling.. Nat Rev Mol Cell Biol.

[30] Swisher EM, Lin KK, Oza AM (2017). Rucaparib in relapsed, platinum-sensitive high-grade ovarian carcinoma (ARIEL2 Part 1): an international, multicentre, open-label, phase 2 trial.. Lancet Oncol.

[31] (2011). Integrated genomic analyses of ovarian carcinoma.. Nature.

[32] Vergote I, Denys H, De Greve J (2020). Treatment algorithm in patients with ovarian cancer.. Facts Views Vis Obgyn.

[33] Vergote I, Gonzalez-Martin A, Ray-Coquard I (2022). European experts consensus: BRCA/homologous recombination deficiency testing in first-line ovarian cancer.. Ann Oncol.

[34] Watkins JA, Irshad S, Grigoriadis A (2014). Genomic scars as biomarkers of homologous recombination deficiency and drug response in breast and ovarian cancers.. Breast Cancer Res.

